# Antiviral RNA Interference against Orsay Virus Is neither Systemic nor Transgenerational in Caenorhabditis elegans

**DOI:** 10.1128/JVI.03664-14

**Published:** 2015-09-23

**Authors:** Alyson Ashe, Peter Sarkies, Jérémie Le Pen, Mélanie Tanguy, Eric A. Miska

**Affiliations:** a Wellcome Trust Cancer Research United Kingdom Gurdon Institute, University of Cambridge, Cambridge, United Kingdom; b Department of Biochemistry, University of Cambridge, Cambridge, United Kingdom; c Department of Genetics, University of Cambridge, Cambridge, United Kingdom; d School of Molecular Bioscience, University of Sydney, Sydney, NSW, Australia; e MRC Clinical Sciences Centre, Imperial College London, London, United Kingdom

## Abstract

Antiviral RNA-mediated silencing (RNA interference [RNAi]) acts as a powerful innate immunity defense in plants, invertebrates, and mammals. In Caenorhabditis elegans, RNAi is systemic; i.e., RNAi silencing signals can move between cells and tissues. Furthermore, RNAi effects can be inherited transgenerationally and may last for many generations. Neither the biological relevance of systemic RNAi nor transgenerational RNAi is currently understood. Here we examined the role of both pathways in the protection of C. elegans from viral infection. We studied the Orsay virus, a positive-strand RNA virus related to Nodaviridae and the first and only virus known to infect C. elegans. Immunity to Orsay virus infection requires the RNAi pathway. Surprisingly, we found that genes required for systemic or transgenerational RNAi did not have a role in antiviral defense. Furthermore, we found that Orsay virus infection did not elicit a systemic RNAi response even when a target for RNAi was provided by using transgenes. Finally, we show that viral siRNAs, the effectors of RNAi, are not inherited to a level that provides any significant resistance to viral infection in the next generation. We conclude that systemic or transgenerational RNAi does not play a role in the defense against natural Orsay virus infection. Furthermore, our data suggest that there is a qualitative difference between experimental RNAi and antiviral RNAi. Our data are consistent with a model of systemic and transgenerational RNAi that requires a nuclear or germ line component that is lacking in almost all RNA virus infections.

**IMPORTANCE** Since its discovery in Caenorhabditis elegans, RNAi has proven a valuable scientific tool in many organisms. In C. elegans, exogenous RNAi spreads throughout the organism and can be passed between generations; however, there has been controversy as to the endogenous role(s) that the RNAi pathway plays. One endogenous role for which spreading both within the infected organism and between generations would be advantageous is a role in viral defense. In plants, antiviral RNAi is systemic and the spread of RNAi between cells provides protection against subsequent viral infection. Here we investigated this by using the only naturally occurring virus known to infect C. elegans, Orsay virus, and surprisingly found that, in contrast to the exogenous RNAi pathway, the antiviral RNAi response targeted against this virus does not spread systemically throughout the organism and cannot be passed between generations. These results suggest that there are differences between the two pathways that remain to be discovered.

## INTRODUCTION

RNA interference (RNAi) is a mechanism of gene silencing that is broadly conserved across eukaryotes. RNAi is initiated by the cleavage of long double-stranded RNA (dsRNA) by the RNase III enzyme Dicer into short 20- to 24-nucleotide (nt) small interfering RNAs (siRNAs) (1). These siRNAs are bound by Argonaute proteins and act as a guide to the complementary mRNA, which is subsequently destroyed by the slicer action of the Argonaute protein (2).

In Caenorhabditis elegans, there is an additional amplification step in the pathway. siRNAs generated by Dicer (DCR-1) form complexes with Argonaute proteins that recruit RNA-dependent RNA polymerases (RdRps) to the target mRNA. The RdRps produce an abundant class of siRNAs (3, 4) that are almost exclusively 22 nt long and possess a guanine (G) as the 5′ nucleotide; hence, they are referred to as 22G RNAs. 22G RNAs are more abundant than siRNAs produced by Dicer and are required for effective gene silencing. However, they are not able to recruit RdRps to the target and thus are unable to initiate the generation of further 22G RNAs (5).

The exogenous RNAi pathway in C. elegans is systemic (6, 7). Uptake of dsRNA into the intestine by the transporter protein SID-2 and transfer between cells by SID-1 and SID-5 are capable of silencing gene expression in most tissues (8–11). The exact mobile RNA species remains elusive, but there is some evidence to suggest that dsRNA molecules (probably DCR-1 products) are mobile and can be exported and imported by proteins required for systemic RNAi (12).

Silencing initiated by the exogenous RNAi pathway can spread not just within treated animals but also to their offspring in what is known as transgenerational inheritance (13–16). Transgenerational inheritance initiated by RNAi does not occur at every locus and is not fully penetrant; i.e., not all offspring inherit the silenced phenotype. The mechanisms responsible for both the transmission and the establishment of transgenerational silencing remain cryptic, although it seems that both small RNA pathways and chromatin modifiers are required (13–18).

RNAi acts as a potent defense mechanism against viruses in plants and animals (19–23). In plants, long viral dsRNA precursors are processed into 21-nt-long siRNAs by Dicer-like 4 (24, 25). These 21-nt siRNAs are capable of moving from cell to cell and directing silencing in the recipient cell (26, 27). The spread of siRNAs ahead of viral infection confers resistance on recipient cells. Twenty-four-nucleotide Dicer-like 3 products are also capable of systemic spreading (26, 28). Similarly, Drosophila also utilizes a siRNA pathway in viral defense. In this case, both Dicer 2 and Argonaute 2 are required for an effective antiviral response (29). Again, systemic spreading throughout the organism is important in antiviral immunity, although in this case it seems to be dsRNA molecules that are mobile (29). RNAi pathways have also recently been suggested to play a role in viral defense in mammals (20, 21), although to date it is not known if the antiviral silencing can spread between cells.

The RNAi pathway also acts in viral defense in C. elegans. The initial trigger is a dsRNA viral replication intermediate that is recognized by DRH-1 (30). This recognition allows DCR-1 and accessory proteins to produce siRNAs and subsequently 22G RNAs in a manner that appears to utilize the same pathway as classical RNAi gene silencing (19, 30–32); indeed, our current knowledge suggests that it is only the viral recognition factor DRH-1 that differs between the two pathways.

Despite the similarities to the canonical RNAi pathway, it is still unclear whether the C. elegans antiviral siRNA pathway gives rise to systemic effects. In the case of a virus that infects somatic cells, such as the Orsay virus, indirect evidence to support systemic antiviral RNAi could be taken from transgenerational inheritance of silencing, since this implies that the RNAi response must have spread into the germ line. Transgenerational silencing of a Flock House virus transgene under the control of a heat shock promoter has been observed (33); however, this may occur as a result of the presence of the transgene in all of the cells of the animal. More recently, it was reported that parental exposure to Orsay virus can protect offspring from infection (34). However, Guo and colleagues reported that the *sid-1* mutant is no more susceptible to Orsay virus infection than N2 is, suggesting that systemic RNAi is not important in viral defense (35).

In this study, we tested for the existence of a systemic RNAi response against the Orsay virus by using a sensor for antiviral siRNA to report on the spread of antiviral silencing between cells. Surprisingly, in contrast to the exogenous RNAi pathway in C. elegans and viral defense pathways in both plants and Drosophila, we found that RNAi following infection with Orsay virus is not systemic. Consistently, we found that there is no transgenerational inheritance of Orsay virus-induced silencing. Together, these results suggest partitioning between intermediates in RNAi induced by the Orsay virus and exogenous dsRNA and challenge the assumption that systemic RNAi evolved as an antiviral defense mechanism.

## MATERIALS AND METHODS

### Genetics.

C. elegans was grown under standard conditions at 20°C on HB101 bacteria unless otherwise indicated. The wild-type strain was var. Bristol N2 (36). The strains used in this study were HC75 [*ccIs4251 sid-1*(*qt2*)], HC271 [*ccIs4251 qtIs3 sid-2*(*qt42*) *mIs11*], RB2519 [*drh-1*(*ok3495*)], SX2836 (*mjIs242*[*psur-5*::*GFP*::*OrsayRNA2*::*tbb-2* + *psur-5*::*mCherry*::*unc-54*]), SX2839 {*mjIs242*[*psur-5*::*GFP*::*OrsayRNA2*::*tbb-2* + *psur-5*::*mCherry*::*unc-54*] *sid-1*(*qt2*)}, SX2838 {*mjIs242*[*psur-5*::*GFP*::*OrsayRNA2*::*tbb-2* + *psur-5*::*mCherry*::*unc-54*] *drh-1*(*ok3495*)}, and SX2813 (*mjEx565*[*psur-5*::*GFP*::*OrsayRNA1*::*tbb-2*] *mjEx566*[*psur-5*::*mCherry*::*unc-54*]).

### Molecular biology. (i) Sensor generation.

The psur-5::GFP::OrsayRNA2::tbb-2 and psur-5::GFP::OrsayRNA1::tbb-2 constructs were generated by using the MultiSite Gateway Three-Fragment Vector Construction kit (Life Technologies). psur-5 in the first position was a gift from the Ahringer laboratory, and green fluorescent protein (GFP; cloned from pPD95.75) was placed in the second position. PCR fusion was used to generate the OrsayRNA::tbb-2 fragments that were placed in the third position. All three fragments were combined in an LR reaction into the pCFJ150 vector. The sequences of the primers used to amplify viral segments from cDNA are available on request.

### (ii) Preparation of RNAi constructs.

Viral segments were PCR amplified from cDNA by using primers with appropriate tails for BP cloning into pDONR221 (Gateway cloning). LR reactions were performed to place viral segments in a Gateway modified RNAi vector L4440. The sequences of the primers used are available on request.

### Detection of viral infection.

Virus filtrate was prepared as described previously (19).

### (i) Infection of strains of interest.

For all strains, two or three young adults were inoculated with 20 μl of viral filtrate for 4 days at 20°C in 55-mm plates.

### (ii) Detection of viral RNA.

Four days after infection, all animals were collected in M9; RNA extraction and quantitative reverse transcription (qRT)-PCR was performed as described previously (19, 30). Aliquots of RNA were kept apart for small RNA libraries (see below).

### (iii) Comparison of infection methods.

For liquid culture-based infection, 200 L2 animals were rotated for 1 h at 20°C in 300 μl of M9, 100 μl of HB101 in LB broth, and 100 μl of Orsay virus filtrate (nondiluted or diluted in M9 10 or 100 times). After 1 h, the animals were collected and washed three times in M9 before transfer to 50-mm plates.

For agar-based infection, 200 L2 animals were transferred to a 50-mm plate per individual infection. A 100-μl volume of Orsay virus filtrate was added (nondiluted or diluted in M9 10 or 100 times). Infections were performed in five biological replicates.

For all strains, 200 L2 animals were infected and collected 48 h postinfection for detection of viral RNA as described above.

### RNAi.

RNAi bacteria were grown for 6 h with shaking at 37°C. Bacteria were then seeded onto 55-mm nematode growth medium plates containing isopropyl-β-d-thiogalactopyranoside (IPTG; 1 mM) and carbenicillin (25 μg/ml). After drying overnight, two or three animals were added and then grown at 20°C for 4 days, at which point they were collected for RNA extraction (for measurement of Orsay viral loads or for small RNA sequencing) or their phenotype was scored (for the viral sensor experiments). For the analysis of phenotypes of animals treated with RNAi against the *unc-22* (ZK617.1) or *dpy-11* (F46E10.9) gene (37), four L4 animals were plated on RNAi plates (five replicates) against the relevant gene and the progeny was grown on the same plates. Adult animals were then transferred to non-RNAi plates, and the phenotype of the progeny was scored to measure the percentage of animals displaying the phenotype. The experiment was repeated in triplicate.

### Small RNA sequencing. (i) RNA extraction, library preparation, and sequencing.

Extraction of RNA for libraries, library preparation, and sequencing were performed as described previously (30). P0 animals were grown at 20°C on three 10-cm plates and collected as a mixed-stage population of predominantly adults 4 days after viral infection or RNAi treatment. F1 animals were obtained by bleaching of P0 animals and assayed at three different ages—as embryos (immediate collection) and after 24 and 72 h. P0 infection was performed in biological duplicates, with one replicate used for the embryos and the other used for both the 24- and 72-h time points. P0 replicates were compared to ensure equality.

### (ii) Sequencing analysis.

Small RNA libraries were sequenced by using Illumina MiSeq and/or HiSeq. Processing and alignment of high-throughput sequencing data to the Orsay virus were carried out as described previously, by using Bowtie for all sequence alignments and allowing up to one mismatch to compensate for divergence in the viral sequence (30). To generate plots of small RNAs aligning to *unc-22* and *dpy-11*, coding sequences in fasta format for the *dpy-11* and *unc-22* genes were downloaded from WormBase (WS236) and used to build genomes with Bowtie-build to which small RNAs were aligned while allowing 0 mismatches.

### Microarray data accession number.

The raw high-throughput functional genomics data obtained in this study are available under GEO accession number GSE60020.

## RESULTS

### Weak transgenerational transmission of antiviral siRNAs.

**FIG 1:**
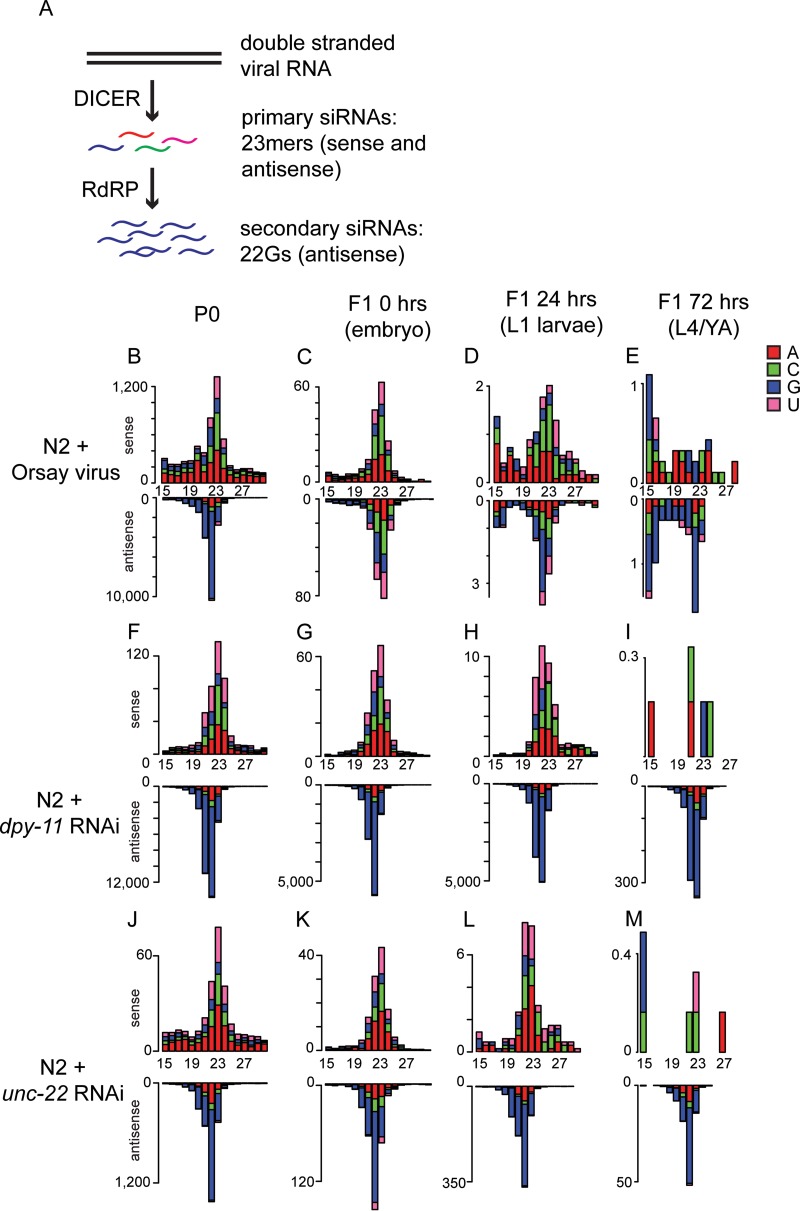
Deep sequencing of viRNAs after Orsay virus infection and RNAi. (A) Cartoon showing the pathway of the 23-nt Dicer product and 22G RNA production in C. elegans. (B to M) 5′ independent small RNA sequencing of P0 and F1 animals after either Orsay virus exposure (B to E) or the RNAi treatment indicated (F to M). P0 animals were assayed as a mixed-stage population of predominantly adults, and F1 animals were synchronized and assayed at three different ages as indicated. Data are shown as sense or antisense and ordered according to the size of the RNA molecule. The values on the *y* axis are reads per million.

Exposing C. elegans to dsRNA through feeding, injection, or viral infection results in the generation of two classes of siRNAs that bring about RNAi (Fig. 1A). The members of the first class of siRNAs are generated by DCR-1 activity on dsRNA, possess 5′ monophosphates, and have a modal length of 23 nt and no overall first-nucleotide bias. The members of the second class of siRNAs (22G RNAs) are generated by the activity of RdRp and have 5′ triphosphates with a strong preference for G at the first nucleotide. Standard small RNA library preparation cannot detect 22G RNAs unless the 5′ triphosphate is removed by enzymatic treatment. We performed polyphosphatase treatment of the RNA before library preparation, which enables the detection of both direct Dicer products and 22G RNAs.

**FIG 2:**
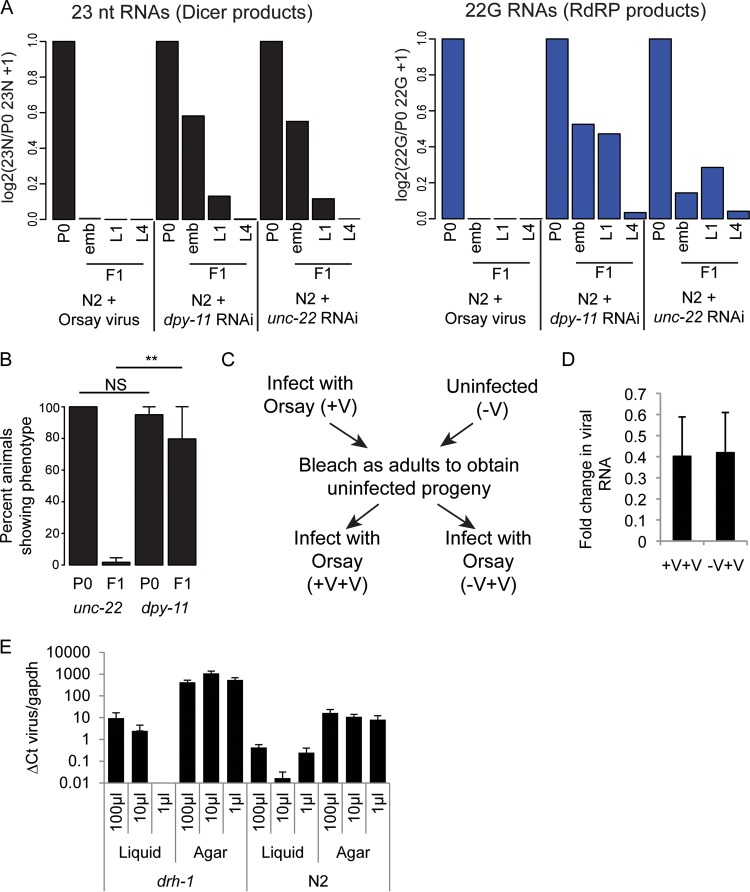
No evidence of inheritance of viRNAs after Orsay virus infection. (A) The 23-nt sense (left) Dicer products and 22G antisense (right) secondary RNAs from Fig. 1 normalized to library size and the level in the P0 generation. (B) Percentages of P0 and F1 animals displaying the *dpy*- or *unc*-encoded phenotype following exposure to RNAi. Error bars show the standard deviation of three (P0) or four (F1) biological replicates. **, *P* < 0.005; NS, not significant (*t* test). (C) The experimental design for the data shown in panel D. (D) qRT-PCR data for the relative levels of Orsay virus 4 days after exposure in animals whose parents were either infected with Orsay virus (+V+V) or uninfected (−V+V) (Orsay virus infection of parents was confirmed by qRT-PCR). Data are normalized to *gapdh* levels and the level of infection of the +V parents. Error bars show the standard error of the mean. (E) Graph showing a comparison of Orsay virus infection levels between liquid- and agar-based infection protocols in both the *drh-1* mutant and N2 strains with three different concentrations of virus. Each condition was performed with five biological replicates, and error bars represent the standard error of the mean.

To better understand the antiviral RNAi response, we used high-throughput sequencing to assess the small RNAs present in biological duplicates of N2 animals infected with Orsay virus (N2 P0) and compared them with their uninfected offspring (N2 F1) (Fig. 1B to E). As shown previously (30), the N2 P0 sample (Fig. 1B) shows Dicer products mapping both sense and antisense to the viral genome and abundant 22G RNAs mapping antisense to the viral RNA. There are considerably less viral interfering RNAs (viRNAs) in F1 animals than in their parents and they decrease over time (Fig. 1C to E and 2A).

To get an idea of how these very low levels of inherited viRNAs compared to inheritance following exogenous RNAi, we also prepared libraries from animals feeding on RNAi food against the endogenous loci *dpy-11* (Fig. 1F to I) and *unc-22* (Fig. 1J to M) and their offspring, which were fed standard HB101 bacteria. Both the *dpy-11* and *unc-22* genes are expressed in somatic cells; however, the *dpy-11* phenotype conferred by RNAi can be inherited by the F1 generation while the *unc-22* phenotype cannot (Fig. 2B) (38). siRNAs complementary to both *dpy-11* and *unc-22* were approximately as abundant as viRNAs in infected P0 animals (Fig. 1F and J). Both *dpy-11* and *unc-22* siRNAs were retained to considerably higher levels than the viRNAs in the F1 offspring (Fig. 2A). Notably, 22G RNAs are still clearly visible in the offspring of both *unc-22* and *dpy-11* mutant-treated animals at all of the time points sampled; *dpy-11* 22G RNA levels were higher than *unc-22* 22G RNA levels, consistent with the inheritance of the *dpy-11* phenotype. Thus, the viral siRNAs are inherited by the F1 generation at a lower level than endogenous, somatically expressed loci, even those for which a phenotype is not detected in the F1 generation.

### No evidence of a protective “vaccination” effect in F1 animals.

The fact that viRNAs appear to be inherited at such low levels strongly suggested that they would be unable to protect the F1 generation against future viral infection. To test this directly, we grew wild-type animals in the presence or absence of virus, “bleached” them to remove infected P0 animals and the Orsay virus from the culture, and then reinfected the F1 generation. If the small number of inherited viRNAs could protect the subsequent generation against viral infection, we would expect to see lower levels of viral replication in the offspring of infected parents than in uninfected parents. We detected viral loads in F1 animals 4 days after infection by quantitative PCR for Orsay virus RNA. There was no difference in Orsay virus RNA levels between the animals whose parents had been exposed to Orsay virus and those with no prior exposure (Fig. 2C and D). These results suggest that the few viRNAs detected in the F1 generation are insufficient to induce viral silencing. This lack of F1 “vaccination” by the Orsay virus contrasts with recently published work by Sterken et al. (34). One main difference between the two studies is the infection procedure—infection in liquid culture for 1 h, followed by growth on agar (Sterken), versus infection and growth on agar (this study). It is plausible that liquid-based infection may result in higher levels of viral infection and thus make F1 “vaccination” possible. To address this issue, we tested the levels of infection produced by the two methods in both N2 and *drh-1* animals and found that agar-based infection was more reproducible and resulted in higher levels of infection than liquid culture-based infection over a range of viral concentrations (Fig. 2E).

### Generation of an antiviral 22G RNA sensor to detect viral infection.

**FIG 3:**
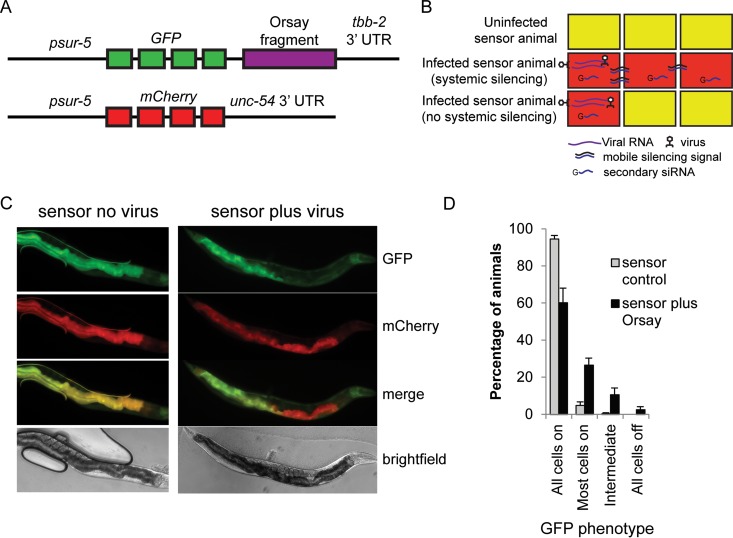
Development of a viRNA sensor. (A) Schematic of viRNA sensor. (B) Cartoon showing the expected phenotype of sensor animals in the absence of Orsay virus (top) and in the presence of Orsay virus with (middle) or without (bottom) systemic silencing. Yellow indicates both GFP and mCherry expression. Red indicates only mCherry expression. (C) Orsay virus sensor showing representative uninfected (left) and infected (right) animals. Infected- and uninfected-animal images were taken at the same intensity. (D) Percentages of sensor animals showing the amounts of silenced cells indicated in the absence (gray) or presence (black) of Orsay virus. Error bars indicate the standard error of the mean of six biological replicates.

To understand the reasons for the failure of viRNAs to be inherited by the F1 generation, we developed a GFP sensor capable of detecting antiviral 22G RNAs. The sensor consists of an integrated, multicopy transgenic array of the ubiquitous *sur-5* promoter driving GFP expression, followed by approximately 600 bp of *OrsayRNA2* before the *tbb-2* 3′ untranslated region (UTR) (*psur-5*::*GFP*::*OrsayRNA2*::*tbb-2*). The animals also carry the same promoter driving mCherry expression with an alternate 3′ UTR (*psur-5*::*mCherry*::*unc-54*) (Fig. 3A). In this system, uninfected animals should express both GFP and mCherry, resulting in “orange” animals. Upon Orsay virus infection, viRNAs produced in the infected cells should silence the GFP transgene, resulting in red cells. As RNAi is systemic in C. elegans, the mobile silencing signal generated after or during the dicing of viral dsRNA should spread systemically through the organism. When, in an uninfected cell, the mobile species encounters the mRNA produced from the sensor transgene, the mobile signal should trigger the production of 22G RNAs and silence the sensor (Fig. 3B).

**FIG 4:**
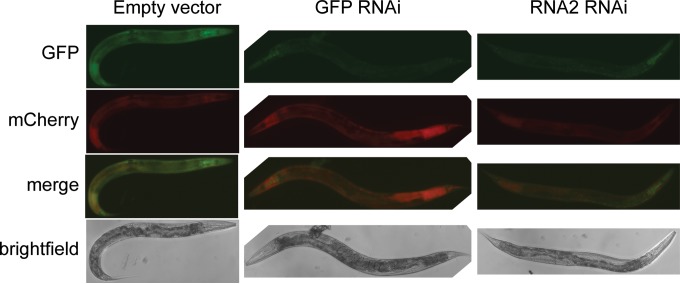
Orsay virus sensor with RNAi. The Orsay virus sensor shows representative animals treated with the RNAi clones indicated. All images were taken at the same intensity.

GFP and mCherry expression in uninfected animals is ubiquitous but predominately intestinal (Fig. 3C). To confirm that the sensor is responsive to silencing in all cells, we performed RNAi against GFP or against the *OrsayRNA2* fragment. This treatment silences the sensor robustly in all animals, with residual GFP expression in the pharynx only (the pharynx is known to be somewhat RNAi resistant) (Fig. 4), confirming that exogenous RNAi silences this sensor systemically. Surprisingly, however, when we exposed these sensor animals to the Orsay virus, GFP silencing was not observed systemically; although 40% of the animals displayed at least one silenced intestinal cell, less than 3% failed to silence all intestinal cells (Fig. 3D).

### Orsay virus infection does not produce a mobile siRNA signal.

**FIG 5:**
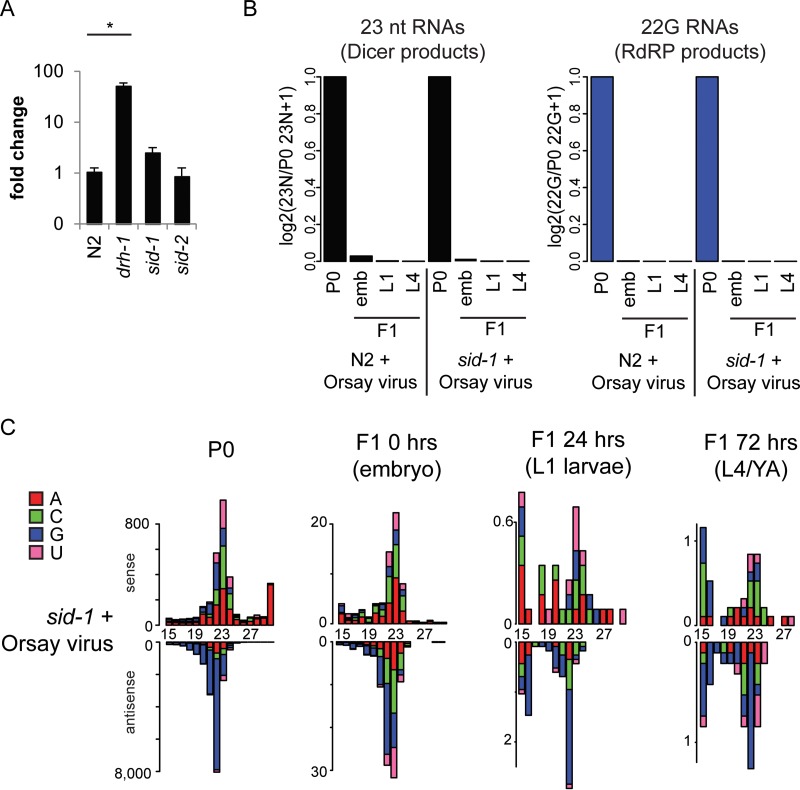
*sid-1* and *sid-2* are not required for viral resistance. (A) qRT-PCR showing the relative levels of Orsay virus 4 days after infection in N2 and in *drh-1*, *sid-1*, and *sid-2* mutants. *drh-1* mutant animals show significantly higher levels of Orsay virus RNA than N2 animals do (*P* < 0.05, *t* test), but there is no significant difference between either *sid-1* or *sid-2* mutant and N2 animals. Data were normalized to *gapdh* and then N2. (B) Shown are the 23-nt sense (left) Dicer products and 22G antisense (right) secondary RNAs from panel C (*sid-1*) and Fig. 1 (N2) normalized to library size and the level in the P0 generation. emb, embryo. (C) 5′ independent small RNA sequencing of P0 and F1 *sid-1* mutant animals after Orsay virus exposure. P0 animals were assayed as a mixed-stage population of predominantly adults, and F1 animals were synchronized and assayed at three different ages as indicated. Data are shown as sense or antisense and ordered according to the size of the RNA molecule. The values on the *y* axis are reads per million. The 5′ nucleotide is indicated by color as follows: red, A; green, C; blue, G; pink, U.

Given the limited ability of the antiviral RNAi response generated against the Orsay virus to spread between cells, we hypothesized that systemic RNAi may not be important in defending against viral infection. Supporting this view, Guo et al. recently reported results suggesting that, indeed, Orsay virus accumulated to similar levels in N2 and a *sid-1* mutant (35). We confirmed this result and additionally showed that *sid-2* is dispensable for viral defense (Fig. 5A). In order to further study the role of *sid-1* in viral defense, we assayed the siRNAs present in Orsay-infected animals and their offspring by small RNA sequencing as described above for N2 animals (Fig. 5B and C). We could detect no difference in small RNAs between the *sid-1* mutant strain and N2, further confirming that *sid-1* transport of mobile siRNAs is not required for their generation in the context of Orsay virus infection.

**FIG 6:**
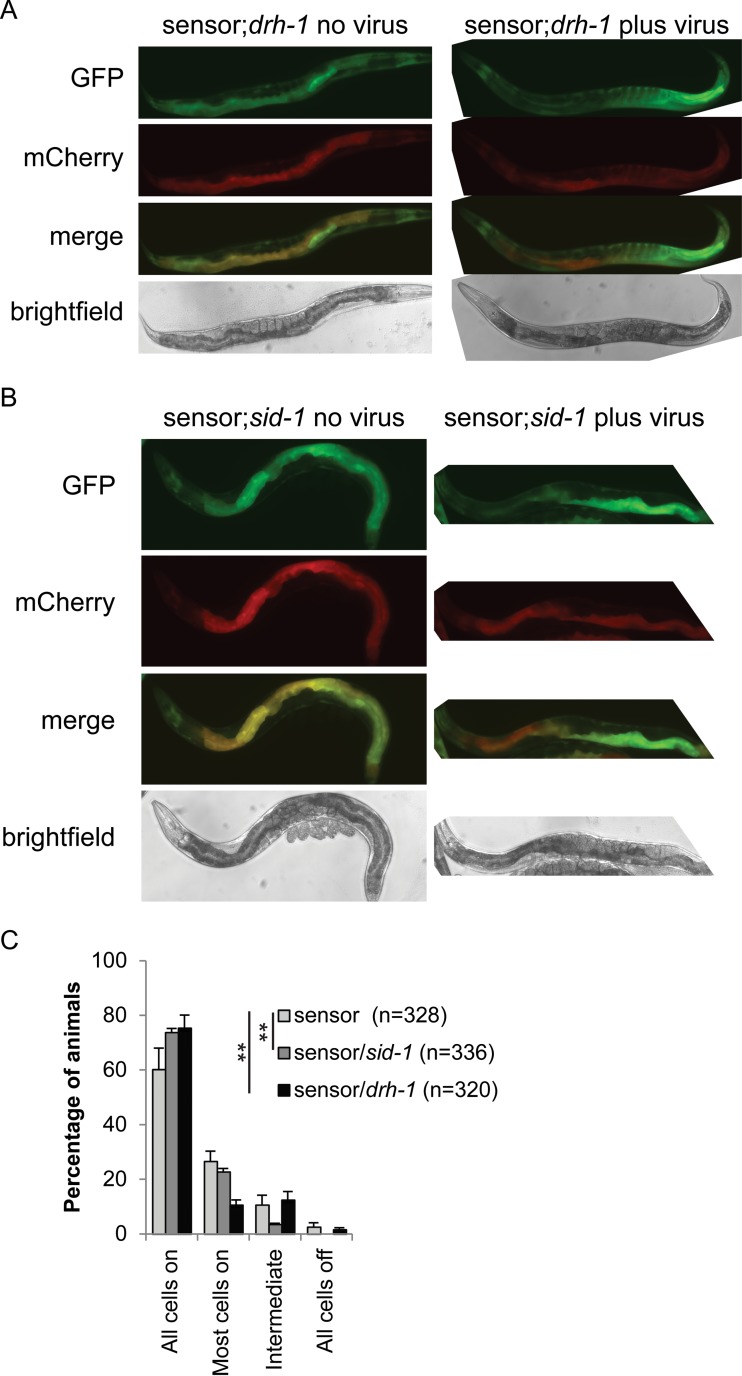
Orsay virus sensor in the *sid-1* and *drh-1* mutant backgrounds. (A, B) Orsay virus sensor showing representative uninfected (left) and infected (right) animals. Infected- and uninfected-animal images were taken at the same intensity. (C) Percentages of sensor N2 (light gray), *sid-1* mutant (gray), and *drh-1* mutant (black) animals showing the amounts of silenced intestinal cells indicated in the presence of Orsay virus. The amount of intestinal cell silencing differs significantly between both the *sid-1* and *drh-1* mutant backgrounds and the wild-type background (*P* < 0.001 [Fisher's exact test] in both cases). Error bars represent the standard error of the mean of six biological replicates.

If RNAi is not moving from cell to cell during infection with the Orsay virus, cases where we observe sensor silencing in more than one cell would occur only because the virus directly infects each cell. To test this hypothesis, we crossed the sensor into the *drh-1* mutant background, which displays increased susceptibility to viral infection (Fig. 6A). *drh-1* mutant sensor animals displayed an increased number of animals with many silenced cells (Fig. 6C), suggesting that silencing of the sensor is driven by infection rather than mobile RNAi. While *drh-1* mutant animals are defective in the production of antiviral siRNAs, they do produce them at low levels (30), enough to silence the sensor in infected cells.

**FIG 7:**
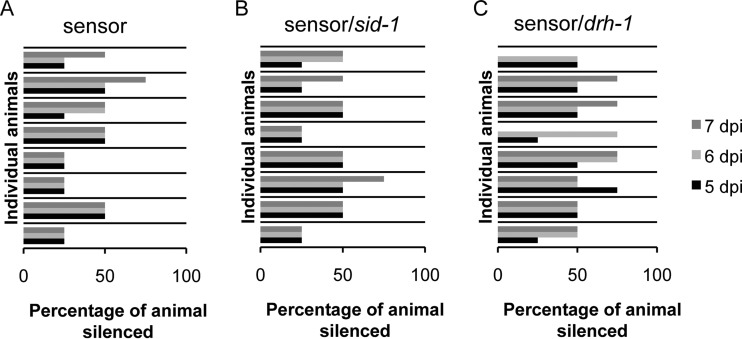
No systemic sensor silencing following Orsay virus infection. Horizontal blocks indicate individual infected animals monitored for 3 days at 5 (black), 6 (light gray), and 7 (dark gray) days postinfection. The values on the *x* axis are the percentages of the animal silenced. Eight N2 (A), *sid-1* mutant (B), and *drh-1* mutant (C) animals each were monitored.

To test the lack of cell-to-cell spreading further, we crossed the sensor into the *sid-1* mutant background. As the *sid-1* mutant is as susceptible to viral infection as N2 is (Fig. 5A), there should be no difference between the number of silenced cells within individual animals between N2 and *sid-1* mutant sensor animals. However, when we crossed the sensor into *sid-1* mutants (Fig. 6B), there was a significant difference between the numbers of animals with multiple silenced cells in the *sid-1* mutant and N2 backgrounds (Fig. 6C). This might indicate that a low level of spreading of silencing from one cell to its direct neighbor does occur in N2. To address this issue in all three strains, we monitored eight partially silenced animals individually over 3 days and the GFP silencing never became systemic (Fig. 7). Over the 3 days that we monitored the N2 and *sid-1* mutant sensor animals, the number of GFP-silenced cells rarely changed, indicating that if a silencing signal passes from one cell to another, it happens very slowly or infrequently. In the *drh-1* background, the number of silenced cells increased in more than half of the animals over the 3-day period. Although not significantly different from N2, this trend is consistent with an increased number of infected cells due to the higher levels of infection known to be sustained in this background (30).

Taken together, these results indicate that, unlike in exogenous RNAi, viral infection does not result in large numbers of dsRNA intermediates able to be trafficked by SID-1.

### Orsay-derived transgenes are not sufficient to enable inheritance of antiviral siRNAs against the Orsay virus.

The lack of inheritance of the antiviral RNAi response against the Orsay virus was surprising given that Flock House virus transgene silencing was reported to be transgenerationally inherited (33). One explanation for this discrepancy could be that the Flock House virus transgene is carried in all cells, while the Orsay virus infects only intestinal cells. Transgenerational silencing might therefore not occur in the Orsay virus because there is a requirement for a template, either RNA or DNA, in the germ line in order to transmit a silencing signal. To test this, we asked whether Orsay virus infection could transmit silencing of the viral sensor transgene, carried in all cells, to the next generation.

**FIG 8:**
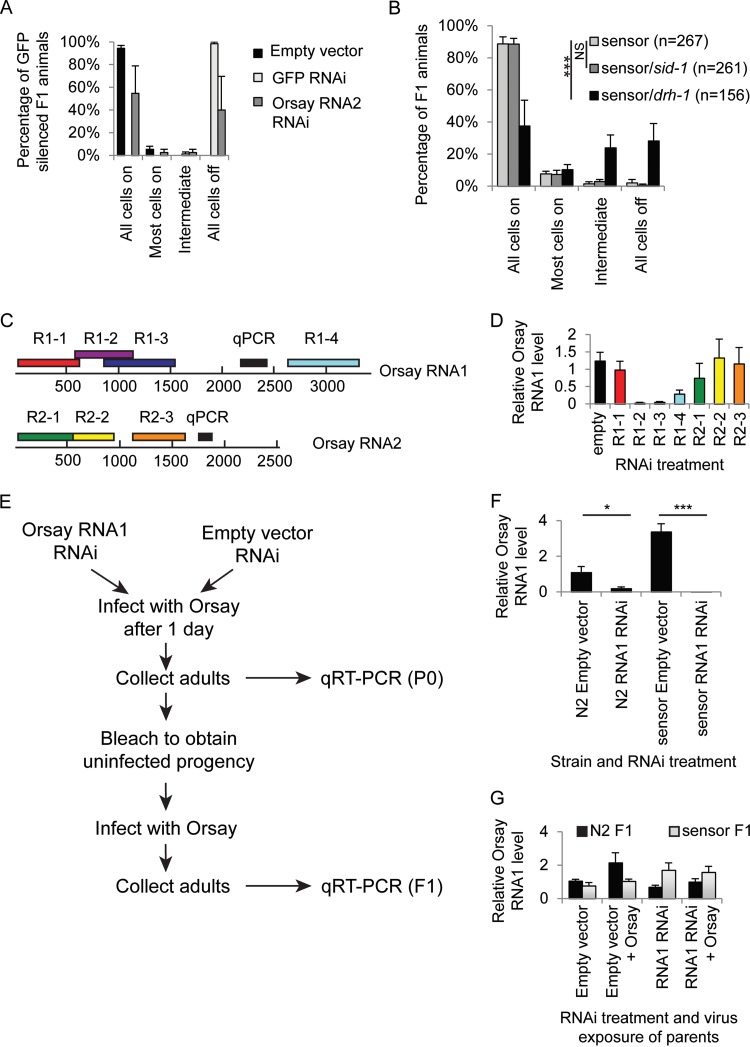
RNAi-induced Orsay virus silencing. (A) Graph showing the percentages of F1 animals with inherited sensor silencing after parental exposure by feeding to RNAi against either the empty vector (black), GFP (light gray), or *OrsayRNA2* (dark gray). Error bars represent the standard error of the mean of two biological replicates. (B) Sensor silencing in the F1 offspring of infected animals (as judged by sensor silencing). The values on the *x* axis are the percentages of N2 (light gray), *sid-1* mutant (dark gray), and *drh-1* mutant (black) animals with the indicated amounts of sensor silencing in the intestine. ***, *P* < 0.0005 (Fisher's exact test). (C) Cartoon showing the position on the Orsay virus genome of the RNAi clones used in panel D and the positions of the quantitative PCR (qPCR) amplicons. (D) Graph showing the relative levels of Orsay virus (4 days postinfection) measured by qRT-PCR in N2 animals fed the RNAi clones indicated. Error bars represent the standard error of the mean of 3 (R1-1, R1-2, R2-1), 6 (R1-3, R1-4, R2-2, R2-3), or 12 (empty) biological replicates. (E) Schematic illustrating the experimental design used to test for the presence or absence of viral resistance in the F1 generation caused by previous viral exposure, viral RNAi, or both. (F) Graph showing the relative Orsay virus levels measured by qRT-PCR in P0 animals 4 days after Orsay virus exposure. Animals were wild type or carried an *OrsayRNA1* sensor transgene and were exposed to either the empty vector or *OrsayRNA1* RNAi. Exposure to Orsay virus RNAi in the P0 generation causes resistance to Orsay viral infection in both genetic backgrounds, although the effect is more significant in the sensor background. Data were normalized to *gapdh* and then the N2 empty vector. *, *P* < 0.05; ***, *P* < 0.0005 (*t* test). Error bars represent the standard error of the mean of three biological replicates. (G) Relative *OrsayRNA1* levels in F1 animals. The *x* axis shows the RNAi treatment and/or Orsay exposure of their parents. N2 animal are shown in black, and RNA1 sensor animals are in light gray. There is no significant difference in any treatment or strain. Data were normalized to *gapdh* and then the N2 F1 empty vector. Error bars represent the standard error of the mean of three biological replicates.

First, we tested whether RNAi induced silencing against either GFP or the *OrsayRNA2* fragment in the P0 generation could result in transgenerational sensor silencing. We subjected P0 *OrsayRNA2* sensor animals to RNAi treatment with either GFP or *OrsayRNA2*. Either treatment resulted in complete sensor silencing, as described previously (Fig. 4). Adults were then removed from the RNAi treatment, and the phenotype of the resultant F1 progeny was scored after 4 days. Both treatments resulted in silencing of the GFP transgene in the F1 generation (Fig. 8A), showing that the sensor is capable of being silenced in a transgenerational manner.

To test for transgenerational silencing of the sensor following Orsay virus exposure, we scored the GFP status of the offspring of eight animals that themselves displayed partial sensor silencing (and were thus infected with Orsay virus). Orsay virus is not transmitted vertically (19), so the only virus present on these plates is that carried by the parent. We performed this experiment in the wild-type sensor and *sid-1* and *drh-1* mutant genetic backgrounds. The offspring of wild-type- and *sid-1* mutant-infected parents showed almost no GFP silencing (and were not significantly different from each other), whereas *drh-1* mutant F1 animals showed significantly more GFP silencing than the wild-type sensor (*P* < 0.001) (Fig. 8B). These data are consistent with no inheritance of transgene silencing following viral infection in any genotype and horizontal transmission of viral infection and thus *de novo* sensor silencing in the case of *drh-1*.

### No protection against infection with the Orsay virus in the F1 generation following RNAi.

We have shown that exposure of parents to the Orsay virus does not confer resistance in F1 progeny and that Orsay virus-induced sensor silencing does not seem to be inherited. RNAi-induced silencing against the viral sensor can however be passed on to the F1 generation; therefore, we asked whether RNAi-induced silencing in the presence of the Orsay virus sensor could protect the P0 generation or F1 offspring against Orsay virus infection.

We tested a series of regions of the Orsay virus RNA genome for the ability to protect against Orsay virus replication in RNAi feeding experiments (Fig. 8C). Intriguingly, Orsay virus levels were affected more after RNAi against *OrsayRNA1* than after RNAi against *OrsayRNA2* (Fig. 8D). This difference in RNAi efficacy between RNA1 and RNA2 could be due to the fact that RNA1 encodes the RdRp and RNA2 encodes the capsid protein. RNAi against the RdRp is more likely to have a direct effect on viral RNA accumulation within infected cells than RNAi against the capsid, which is more likely to affect later steps of the viral life cycle such as assembly.

We then asked whether there was also a reduction in Orsay virus RNA levels following concurrent RNAi and viral infection in N2 or an Orsay virus sensor background (Fig. 8E). Because viral levels were most significantly altered in animals feeding on RNAi clones targeting RNA1 rather than in animals feeding on RNAi clones targeting RNA2, for this experiment, we used an *OrsayRNA1* sensor instead of the previously used *OrsayRNA2* sensor. The sensor was extrachromosomal instead of integrated but otherwise identically constructed. Interestingly, while Orsay virus RNAi reduced the levels of Orsay virus RNA detected in N2, the presence of the RNA1 sensor resulted in even more RNAi-induced protection against Orsay virus replication (Fig. 8F). It is tempting to speculate that this sensor-associated RNAi “boost” is due to the presence of siRNA molecules (generated from the sensor) already in the cell before viral entry, thus enabling immediate viral RNA destruction.

To test whether the combination of RNAi against the Orsay virus and the sensor could confer protection on the F1 offspring, we bleached the adults from the previous experiment to generate uninfected embryos and then infected them with the Orsay virus (Fig. 8E). Despite the large difference in Orsay virus infection levels between RNAi-treated and nontreated sensor animals in the P0 generation, there was no difference in the infection levels of their offspring (Fig. 8G, gray). There was also no difference in viral infection levels in the N2 F1 offspring of parents fed on either empty or RNA1 interfering RNA (Fig. 8G, black).

These data show that even in the most extreme case of RNAi and a transgenic viral portion, there is still no evidence of deposition in the F1 offspring of functional small RNA molecules that can protect against Orsay virus infection.

## DISCUSSION

Two of the most notable aspects of the siRNA pathway in C. elegans initiated in response to exposure to dsRNA matching endogenous genes are its ability to spread throughout the animal and its ability to act transgenerationally. There has been much speculation on what the function of these properties is for animals in the wild, with the proposed role of RNAi in antiviral silencing a key candidate. Here we have shown that infection of C. elegans with the Orsay virus instigates neither transgenerational nor systemic silencing. In the absence of any other known naturally occurring C. elegans virus, it is possible that systemic RNAi may function in defense against an as-yet-undiscovered infection. Nevertheless, our results have important implications for both the mechanism of systemic RNAi and the biology of small RNA pathways in C. elegans.

The fact that antiviral siRNA induced by Orsay virus infection, in contrast to siRNA induced by exposure to dsRNA, does not spread between cells may be explained by differences in the intermediates produced by the two pathways. Importantly, 22G RNAs, produced by both pathways, are unlikely to transfer RNAi in C. elegans (12), perhaps because they cannot themselves trigger the production of further 22G RNAs in somatic cells (5, 39). However, there is evidence that a small RNA species generated by the activity of Dicer/RDE-4 in response to dsRNA generated from endogenous genes or taken up from the environment is able to spread between cells (12). Both RDE-4 and Dicer are active on viral dsRNA; thus, their activity must somehow be different when they act on viral dsRNA rather than other sources of dsRNA. It is possible that this difference is due to the requirement of DRH-1 specifically for activity on viral dsRNA, perhaps reflecting a different subcellular localization of the DRH-1/Dicer complex. An alternative possibility is that the Orsay virus itself prevents systemic RNAi. Such a situation is well known to occur in plant viruses, many of which encode suppressors of silencing that prevent cell-to-cell spreading of silencing (40). Further work is required to distinguish between these two possibilities.

Our observations on the nonsystemic nature of the antiviral RNAi response to infection with the Orsay virus are fully consistent with the facts that we detected no transgenerational protection against infection and did not detect a strong small RNA signal mapping to the virus in the embryos derived from infected individuals. This is in contrast to the robust inheritance of both small RNAs and the silencing phenotype in the case of RNAi-induced silencing of the *dpy-11* gene and inheritance of small RNAs, albeit without an observable phenotype, in silencing of the *unc-22* gene. The lack of systemic RNAi after infection with the Orsay virus thus prevents small RNAs from entering embryos to a sufficient extent to transmit the silencing effect.

The function of RNAi in antiviral defense in plants, fungi, and animals, including mammals, has led to the proposal that viral infection was the major driving force behind its evolution. Systemic silencing, which potentially allows antiviral siRNAs to move ahead of the spread of virus, and transgenerational vaccination of the next generation might seem to be ideal components of an effective antiviral pathway. It is therefore interesting that neither of these two aspects of the C. elegans RNAi pathway is employed in targeting the Orsay virus, despite an absolute requirement for cell-autonomous RNAi in antiviral defense. It remains possible that other naturally occurring viruses will be discovered that can instigate systemic or transgenerational responses, in particular, viruses that could infect the germ line or DNA viruses, which, in plants, appear to be targeted by transgenerational silencing through RNA-directed DNA methylation. It is also possible that, as noted above, the Orsay virus itself has evolved to prevent systemic silencing, although it is worth noting that RNAi-competent strain N2 is unlikely to be the natural host of the Orsay virus. The Orsay virus was discovered in strain JU1580, which is deficient in antiviral RNAi (19, 30); thus, there may not have been strong selective pressure to evolve resistance to systemic silencing in this virus. However, it remains an interesting possibility that systemic RNAi in C. elegans evolved for completely different reasons, linked potentially to its unusual ability to take up dsRNA from its environment. The answers to these questions require deeper sampling of C. elegans in its natural environment to understand better the selective forces acting on the RNAi pathway in the wild.
